# Methanol Extract from *Ranunculus repens* L. Down-Regulated Galectins 4 and 9, and Mitigated Chronic Pancreatitis in an Experimental Rat Model

**DOI:** 10.3390/antiox14121436

**Published:** 2025-11-28

**Authors:** Amir Khenchil, Hocine Rechreche, Arbia Abbes, Elisa Pettineo, Chiara Dianzani, Moufida Bensam, Widad Sobhi, Stefania Pizzimenti

**Affiliations:** 1 Molecular and Cellular Biology Laboratory (MCBL), Faculty of Natural and Life Sciences, University of Jijel, Jijel 18000, Algeria; amir.khenchil@univ-jijel.dz (A.K.); a_abbes@univ-jijel.dz (A.A.); bensamoufida@univ-jijel.dz (M.B.); 2 Department of Clinical and Biological Sciences, University of Turin, Corso Raffaello 30, 10124 Turin, Italy; elisa.pettineo@unito.it; 3 Department of Drug Science and Technology, University of Turin, Via Pietro Giuria 9, 10124 Turin, Italy; chiara.dianzani@unito.it; 4 Biotechnology Research Centre (CRBT), Ali Mendjli UV 03 BP E73, Constantine 25016, Algeria; w.sobhi@crbt.dz

**Keywords:** chronic pancreatitis, *Ranunculus repens* L., oxidative stress, inflammation, fibrosis, galectins 4 and 9

## Abstract

Chronic pancreatitis (CP) is a progressive fibro-inflammatory disease in which oxidative stress (OS) promotes pancreatic stellate cells activation and fibrosis. *Ranunculus repens* L. (*R. repens*) has been used in Algerian traditional medicine to treat conditions like hepatitis and diabetes. Galectins are β-galactoside-binding lectins implicated in several pathological processes, including inflammation. This study aimed to analyse the chemical composition and evaluate the protective effects of *R. repens* methanol extract (RRME) in an experimental CP model, as well as in cultured pancreatic cells. CP was induced by intraperitoneal injections of L-arginine in rats. The pancreas was examined histopathologically, using hematoxylin and eosin, and picrosirius red staining. OS markers were assessed in pancreatic homogenates, and RT-qPCR analysis was performed to evaluate the expression of fibrosis markers, proinflammatory cytokines, and galectins 4 and 9. The extract was characterized by Ultra-performance liquid chromatography mass spectrometry, and its antioxidant and antiapoptotic activities were evaluated in vitro using H_2_O_2_-induced intracellular reactive oxygen species (ROS) generation and paclitaxel-induced apoptosis in pancreatic cell lines. The results showed that treatment with RRME improved relative pancreatic weight and lowered serum lipase activities. It mitigated oxidative stress in pancreatic tissues and reduced fibrosis levels. Inflammation was attenuated, as indicated by decreased interleukin-6, tumor necrosis factor alpha, and leukocyte infiltration. Moreover, RRME down-regulated galectins 4 and 9. Finally, RRME attenuated ROS generation and apoptosis in vitro. These findings suggested that RRME may have therapeutic potential against CP by modulating OS and fibrosis.

## 1. Introduction

Chronic pancreatitis (CP) is a fibro-inflammatory condition that affects the pancreas [1]. It is characterized by ductal changes, significant damage to the exocrine and endocrine pancreas, and progressive fibrosis [2]. Alcohol abuse and smoking are considered the main risk factors for CP. Additionally, metabolic disorders and hereditary factors such as mutations in trypsin1 (PRSS1) also contribute significantly to disease development [3]. The main clinical features of CP include repeated upper abdominal pain, abnormal serum or urine pancreatic enzyme concentrations, abnormal exocrine function, pancreatic duct stones, and parenchymal calcification [4]. CP is often accompanied by a significant impact on life quality [5]. Furthermore, the progressive nature of CP increases the risk of pancreatic cancer [6,7]. With no therapy yet proven effective against CP, there is a critical need for the development of therapeutic strategies to stop CP progression and promote pancreatic tissue regeneration.

Oxidative stress (OS), premature activation of digestive enzymes, and recurrent episodes of acute pancreatitis lead to a progressive inflammatory process causing significant damage to the pancreas [8,9]. This process is accompanied by the recruitment of inflammatory cells, secretion of proinflammatory cytokines, and production of reactive oxygen species (ROS), which are implicated in the activation of quiescent pancreatic stellate cells (PSCs). PSCs transform into a myofibroblast-like phenotype characterized by the expression of alpha-smooth muscle actin (α-SMA) [10]. After activation and proliferation, PSCs play an important role in the deposition of extracellular matrix (ECM) proteins and profibrotic molecules, leading to pancreatic fibrosis [11]. Thus, the inhibition of PSC activation may help prevent fibrosis and slow CP progression.

Galectins are β-galactoside-binding animal lectins classified into three groups based on the number and organization of their conserved carbohydrate-recognition domains (CRDs): the prototype group (e.g., galectin-1), the chimera-type group (e.g., galectin-3), and the tandem-repeat group, which includes galectin-4 (Gal-4) and galectin-9 (Gal-9) [12,13]. Members of this family play various roles in both physiological and pathological conditions, including fibrosis, cell migration, cell differentiation, angiogenesis, and immune responses [14]. Several studies have demonstrated increased expression levels of galectin-1 and galectin-3 in patients with CP [15,16] and pancreatic cancer tissues [17,18]. Moreover, high levels of galectin-1 or galectin-3 have been shown to significantly enhance cell proliferation and migration, as well as increase the expression of ECM molecules [19,20]. Both Gal-4 and Gal-9 are expressed in patients with pancreatic cancer [21,22,23]. They have multifaceted roles in the inflammatory process; Gal-4 promotes intestinal inflammation and stimulates interleukin-6 (IL-6) expression [24] while also contributing to its regulation [25]. On the other hand, Gal-9 contributes to the suppression of allergic lung inflammation [26], and high serum levels of this protein were associated with liver fibrosis in patients with chronic liver disease [27]. Furthermore, Gal-9 was reported to regulate the expression of inflammatory cytokines in monocyte cells [28]. While galectin-1 and galectin-3 have been widely studied in inflammation [29,30], the roles of tandem-repeat galectins such as gal-4 and gal-9 remain poorly understood in chronic pancreatitis. Exploring gal-4 and gal-9 may uncover novel mechanisms of disease progression and potential therapeutic targets.

Phytochemicals from various plants have demonstrated promising potential as novel antifibrotic agents by targeting activated PSCs in cases of CP and pancreatic cancer [31]. The *Ranunculus* genus, belonging to the family Ranunculaceae, encompasses 600 species [32]. Traditionally, plants from this genus have been utilised for their anthelmintic properties, antihaemorrhagic effects, the treatment of asthma, intermittent fevers, diarrhoea, and tympany [33]. Recent research indicates that some members of this genus also possess antioxidant [34], anti-inflammatory [35,36], anticancer [37] and antifibrotic properties [38]. *Ranunculus repens* L. (*R. repens*), a perennial herb distributed in Europe, North Africa, and Asia, is traditionally used for its antihemorrhagic activity [39]. It is used to treat hepatitis and diabetes in Algerian traditional medicine [40]; it also exhibits antibacterial and urease-inhibiting activities [41,42]. However, research on this plant remains limited.

In the present study, we aimed to assess the protective effect of *R. repens* methanol extract (RRME) on L-arginine (L-Arg)–induced CP and to evaluate the expression of Gal-4 and Gal-9 in this context. Although previous studies have highlighted the antioxidant activity of *R. repens* [40], its anti-inflammatory and antifibrotic potential in CP, as well as its phytochemical composition, have not been investigated. Moreover, the expression and functional significance of galectins, Gal-4 and Gal-9, remain unexplored in this context. We hypothesize that RRME may exert protective effects in CP by modulating oxidative stress and inflammatory responses, and that the expression profiles of Gal-4 and Gal-9 may be altered, thereby influencing inflammatory and fibrotic pathways. Accordingly, the specific objectives of this study were to evaluate the antioxidant, anti-apoptotic, and antifibrotic activity of *R. repens*, and to investigate its impact on the expression of Gal-4 and Gal-9 in chronic pancreatitis.

## 2. Materials and Methods

### 2.1. Chemicals and Reagents

Thiobarbituric acid, trichloroacetic acid, Tris, pyrogallol, bovine serum albumin (BSA), 5,5′-dithiobis (2-nitrobenzoic acid) (DTNB), 1-chloro-2,4-dinitrobenzene (CDNB) ascorbic acid, vanillic acid, catechin, rutin, caffeic acid, quercetin, salicylic acid, vanillin, chrysin 8-hydroxyquinoline, thymol, salicin, oleanolic acid, β-carotene and m-Xylene, RPMI 1640 culture medium were obtained from Sigma-Aldrich (St. Gallen, Switzerland). Methanol, hexane, ethyl acetate, n-butanol and hydrochloric acid were acquired from Honeywell (Seelze, Germany). Dimethyl sulfoxide (DMSO), 3-(4,5-dimethyl thiazol-2-yl)-2,5-diphenyltetrazoliumbromide (MTT), Dulbecco’s Modified Eagle Medium (DMEM) high glucose, Foetal bovine serum (FBS), penicillin, streptomycin, and trypsin were from Euroclone (Milan, Italy). The XTT kit was purchased from Roche (Basel, Switzerland). L-Arg hydrochloride was obtained from Biochem Chemopharma (Cosne-Cours-sur-Loire, France), ethylenediaminetetraacetic acid (EDTA) from Chem-Lab (Zedelgem, Belgium), potassium chloride and sodium chloride from Prolab Scientific (Laval, QC, Canada), and hydrogen peroxide from Carlo Erba (Cornaredo, Italy). SuperScript IV reverse transcriptase and 2′-7′ dichlorodihydrofluorescein diacetate (DCF-DA) were purchased from Invitrogen (Waltham, MA, USA), while NucleoZol and Power SYBR Green master mix were obtained from Applied Biosystems™ (Foster, CA, USA). All chemicals and reagents were of analytical grade.

### 2.2. Plant Materials

*R. repens* was collected from El-Milia region (Jijel, Algeria), during its flowering season. The identification of the plant was carried out by Dr. M. Sebti from the Laboratory of Biotechnology, Environment and Health, University of Jijel, and a voucher specimen (RR156) was deposited in the herbarium of the National Park of Taza (www.tela-botanica.org/isfan-nn-152202-synthese, accessed on 24 November 2025). The leaves were air-dried in the shade at room temperature, ground into a fine powder using an electric grinder and stored until extraction.

### 2.3. Animals

All animal procedures were performed in accordance with the international guidelines for the use and care of laboratory animals [43]. The animal study protocol was approved and authorized by the Ethics Committee of the Pasteur Institute of Algiers under the Number FPAL0421/03, in accordance with the Algerian Association for Experimental Animal Science (No. 8808/1988) and regulations concerning veterinary activities and animal health protection (No. JORA/004/1988). Adult male Wistar rats (250–350 g) were procured from the Pasteur Institute (Algiers, Algeria). The animals were pathogen-free, and had no prior procedures before the experimental protocol. The animals were acclimatised for 15 days before the start of the experiments. They were kept under standard environmental conditions, with an ambient temperature of 24 ± 2 °C and a 12 h light/dark cycle, and were allowed free access to water and standard laboratory animal feed ad libitum. Humane endpoints were predefined, including inability to eat or drink, severe lethargy, or other signs of distress such as labored breathing.

### 2.4. Preparation of RRME

RRME was prepared through sequential extraction of 100 g of powdered leaves of *R. repens.* The process began with hexane extraction for 48 h at room temperature, repeated three times, and followed by filtration and concentration, using a rotary evaporator. The remaining solid material was then extracted in the same manner, using ethyl acetate and followed by methanol. The methanol extract was selected for our study.

### 2.5. Ultra-Performance Liquid Chromatography Mass Spectrometry Analysis

The analysis of RRME by Ultra-performance liquid chromatography mass spectrometry (UPLC MS/MS) was performed, using a Shimadzu 8040 ultra-high sensitivity platform (Shimadzu, Kyoto, Japan) equipped with UFMS technology, as well as a Nexera XR LC-20AD binary pump (Shimadzu). The separation of the extract constituents was achieved on a Restek Ultra C18 column (3 µm, 150 × 2.1 mm) (Restek, Bellefonte, PA, USA). The mobile phase was composed of solvent A (formic acid 0.1% dissolved in water) and solvent B (methanol 70%): 5 µL of this mixture was injected with a flow rate of 0.2 mL/min. The gradient protocol included different phases: 0–1 min at 85% A, 1–10 min at 5% A, 10–18 min at 5% A, and 18–23 min at 85% A. The electrospray ionization (ESI) conditions were as follows: CID gas, 230 KPs; conversion dynode, −6 Kv; DL temperature, 250 °C; nebulizing gas flow, 3 L/min; heat block, 400 °C; drying gas flow, 10 L/min. The identification of the compounds was achieved using authentic reference standards available in the laboratory, ensuring accurate and reliable results. The UPLC–MS/MS method enabled the detection of precursor and product ions, providing retention time data and corresponding ion transitions for each compound, which facilitated the precise identification of the constituents in the extract.

### 2.6. Chronic Pancreatitis Induction

**Figure 1:**
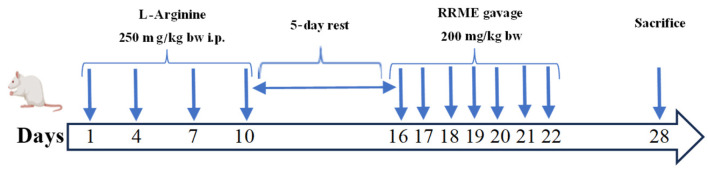
Chronological representation of the L-Arg and RRME administration during chronic pancreatitis induction. Vertical arrows indicate the injection time points, while the last one (day 28) refers to the sacrifice.

Twenty-four animals were randomly assigned to four experimental groups, 6 animals each. The sample size was selected as a practical number, consistent with similar published studies and sufficient to allow for statistical comparisons. The toxicity of RRME was evaluated in an animal model. The extract was well tolerated, with no signs of toxicity or mortality observed at doses up to 2 g/kg body weight, indicating a high safety margin for the concentrations used in this study (data submitted for publication). Based on these findings, a dose of 200 mg/kg was selected for subsequent experiments to ensure both safety and biological activity, as supported by previous reports on *R. repens* leaf extracts [40] and other *Ranunculus* species [35]. CP was induced by repeated intraperitoneal (IP) administration of L-Arg according to the method described by González et al. [44] with some modifications. After overnight fasting, animals received two IP injections with a one-hour interval of 20% L-Arg (pH 7.2) at a dosage of 2.5 g/Kg BW on day 1, followed by single injections on days 4, 7 and 10. A schematic diagram of the experiments is presented in Figure 1. The experimental groups were organized as follows: Group 1 served as the disease control (CP), in which CP was induced as described above. Group 2 served as normal control (C) and received normal saline. Group 3 was the treatment group (RRME + CP) in which the rats received the same treatment as group 1, followed by oral administration with 200 mg/kg BW of RRME daily for one week, five days after the last IP injection of L-Arg. Group 4 received RRME alone (RRME) at a dose of 200 mg/kg BW daily for one week and served as the RRME control. All animals were euthanised on day 28 by cervical dislocation. Blood samples were collected in EDTA tubes. The pancreas was harvested and weighed. One portion of the pancreas was fixed in 10% formalin for histological evaluation and the remaining pancreatic tissues were frozen for biochemical and molecular analysis. The order of treatments and sample collection was balanced across groups, and samples from each group were distributed evenly across assay batches.

### 2.7. Estimation of Plasma Lipase Levels

Serum lipase activity was measured using a lipase kit from Cypress Diagnostics (Hulshout, Belgium) following the manufacturer’s instructions. Briefly, blood samples were centrifuged at 2800 rpm for 20 min, using an Apogee Swing-3000 centrifuge, and plasma was carefully separated with a sterile pipette. Lipase activity was then measured by a SelectraProM automatic analyser (ELI Tech Group, Puteaux, France), and the results were expressed in international units per liter (IU/L).

### 2.8. Tissues Homogenate Preparation

Pancreatic tissues homogenates were prepared following the method described by Iqbal et al. [45]. Briefly, 200 mg of pancreatic tissues were homogenised in 600 µL of cold phosphate buffer (0.1 M, pH 7.4, containing 1.17% potassium chloride), using a TissueLyserRetsch MM 400 (Retsch, Haan, Germany) at 30 Hz for 3 min. The extracts were then centrifuged for 15 min at 4000 rpm and 4 °C, using a Sigma 6–16KS centrifuge (Sigma, Darmstadt, Germany). The supernatant was collected and subjected to a second centrifugation for 30 min at 9000 rpm and 4 °C. The final supernatant was used for the evaluation of OS markers in the pancreas, and the total protein concentration was determined using the Bradford method [46].

### 2.9. Assessment of Lipid Peroxidation

The malondialdehyde (MDA) levels in pancreatic tissues were determined using the thiobarbituric acid (TBA) assay as described by Ohkawa et al. [47], with some modifications. MDA levels were used as an index of lipid peroxidation. Briefly, 125 µL of pancreatic homogenate was mixed with an equal volume of 20% trichloroacetic acid and 250 µL of 0.67% TBA, and then heated in a water bath at 90 °C for 15 min to allow for the development of a pink colour. After cooling, 1 mL of n-butanol was added, and the mixture was centrifuged at 3000 rpm for 15 min, using a Sigma 6–16KS centrifuge. The absorbance of the supernatant was measured using a spectrophotometer at 530 nm, and MDA levels were calculated using the molar extinction coefficient of the TBA-MDA complex (ε = 1.56 × 10^5^ cm^−1^M^−1^). The results were expressed as nmol of MDA per mg of proteins.

### 2.10. Estimation of Reduced Glutathione Content

To estimate reduced glutathione (GSH) levels in pancreatic tissues, we used the method of Ellman [48]. In brief, 5 mL of phosphate buffer (100 mM, pH 8.9) was mixed with 25 µL of pancreatic homogenate. Then, an aliquot of 3 mL from this mixture was mixed with 20 µL of DTNB solution (10 mM) and kept in the dark at room temperature for 5 min. The absorbance was recorded at 412 nm and the GSH concentration was calculated, using the molar extinction coefficient of 14,150 cm^−1^M^−1^. The results were expressed in nmol of GSH per mg of proteins.

### 2.11. Assessment of Pancreatic Antioxidant Enzymes Activities

Superoxide dismutase (SOD) activity in pancreatic tissues was determined following the protocol outlined by Marklund and Marklund [49]. The assay is based on the inhibition of autooxidation of pyrogallol by the enzyme present in the pancreatic homogenate. For this assay, 16 µL of the pancreatic homogenate was mixed with 36 µL of pyrogallol solution (100 mM in 0.01 N HCl), followed by adding 1 mL of Tris-EDTA buffer (50 mM Tris, 1 mM EDTA, pH 8.14). The increase in absorbance was monitored at 430 nm for each sample. One unit of SOD activity was defined as the enzyme quantity needed to inhibit pyrogallol autooxidation by 50%.

The assessment of glutathione S-transferase (GST) activity was carried out following the procedure described by Habig et al. [50]. In summary, a mixture containing 850 µL of phosphate buffer (100 mM, pH 6.5) and 50 µL of CDNB (20 mM) was prepared and incubated at 37 °C for 10 min. The enzymatic reaction was initiated by adding 50 µL of GSH solution (20 mM) and 50 µL of pancreatic homogenate into the mixture. The absorbance was monitored at 340 nm. The activities of SOD and GST were expressed in IU/mg of proteins.

### 2.12. Total RNA Extraction and RT-qPCR Analysis

Total RNA was extracted from pancreatic tissues with the NucleoZol reagent (Applied Biosystems, Foster, CA, USA), following the manufacturer’s instructions. RNA concentration was determined spectrophotometrically at 260 nm, using a NanoDrop™ 8000 Spectrophotometer (Thermo Fisher Scientific, Wilmington, DE, USA). The integrity of the extracted RNA was verified by electrophoresis on a 0.8% agarose gel.

cDNA synthesis was performed, using SuperScript IV Reverse Transcriptase (Invitrogen, Waltham, MA, USA), following the manufacturer’s instructions. PCR amplification was conducted, using Power SYBR Green PCR Master Mix (Applied Biosystems, Foster, CA, USA) following the manufacturer’s instructions. The specific primers for Gal-4, Gal-9, tumour necrosis factor-alpha (TNF-α), IL-6, α-SMA, collagen III (Col-III), and glyceraldehyde 3-phosphate dehydrogenase (GAPDH) are listed in Table 1. The PCR reactions were carried out, using the QuantStudio™ 5 Real-Time PCR System (Applied Biosystems, Waltham, MA, USA) with the following amplification conditions for each reaction: 95 °C for 10 min, followed by 40 cycles of 95 °C for 15 s and 57 °C for 60 s. GAPDH was used as an internal control to normalise the expression of target genes. The relative expression of the target genes was calculated using the 2^−ΔΔCT^ method.

### 2.13. Histopathological Study

To evaluate the effect of RRME on histological alterations in the pancreas, the pancreatic tissues were fixed in 10% buffered formaldehyde, followed by alcohol dehydration and embedding in paraffin. Sections of 5 µm thickness were prepared and stained with haematoxylin and eosin for examination. Structural changes were examined using a modified scoring system based on the method described by Tasci et al. [51]. Leukocyte infiltration, sublobular and lobular atrophy were evaluated under a light microscope in a blinded manner and assigned a score for each criterion ranging from 0 to 3. The sum of these scores constituted the histological score.

Picrosirius red staining was performed to evaluate collagen deposition. Briefly, the tissues sections were deparaffinized and rehydrated, then stained with the staining solution for 1 h, followed by rinsing with acidified water and dehydration with isopropanol for 5 min. For each sample, the positively stained area was quantified using ImageJ software (version 1.52v, National Institutes of Health, Bethesda, MD, USA).and used to represent pancreatic fibrosis.

### 2.14. Cell Lines Culture

Mia PaCa-2, PANC-1 human pancreatic cancer cell lines and mouse pancreatic beta cells (NIT1) were purchased from ATCC (Manassas, VA, USA). Mia PaCa-2 cells were used at passage 7, PANC-1 cells at passage 12, and NIT1 cells at passage 8. Mia PaCa-2 and PANC-1 cells were cultured in DMEM high-glucose medium supplemented with 10% FBS, while NIT1 cells were cultured in RPMI medium supplemented with 15% FBS. All cells were maintained at 37 °C in a humidified atmosphere containing 5% CO_2_, with 100 units/mL penicillin and 100 µg/mL streptomycin.

### 2.15. Measurement of the Cells Redox Status In Vitro

The OS level in MiaPaCa-2 and PANC-1 cell lines was evaluated using DCF-DA as described by Grattarola et al. [52]. Briefly, 300,000 cells were seeded in 6-well plates, then treated with 10 µg/mL or 25 µg/mL of RRME and 200 µM H_2_O_2_ for 24 h at 37 °C. Untreated cells and cells treated with H_2_O_2_ alone served as controls. After treatment, the cells were incubated with 1 µM of DCF-DA for 30 min at 37 °C, and the level of the fluorescent product 2′,7′-dichlorofluorescein (DCF) was quantified using flow cytometry (Becton Dickinson Italia, Milan, Italy).

### 2.16. Annexin V/Propidium Iodide Staining Test

To evaluate the effect of RRME on apoptosis in Mia PaCa-2 cells, we employed the annexin V/propidium iodide (PI) assay. Briefly, 300,000 cells were seeded in 6-well plates and treated with the RRME (100 µg/mL), paclitaxel (400 nM), or a combination of both, untreated cells served as the control. The cells were incubated for 24 h. Both adherent and floating cells were collected, pooled, and centrifuged. The pellets were resuspended in 100 µL of binding buffer, then stained with annexin V and PI according to the manufacturer’s instructions, and the cells were analysed using a flow cytometer (Becton Dickinson Italia, Milan, Italy).

### 2.17. Statistical Analysis

In all experiments, the statistical analyses and tests for data normality were carried out using GraphPad Prism version 8.0.2 (263). The results were expressed as mean values with the corresponding standard error of the mean. To assess differences among various groups, a one-way analysis of variance (ANOVA) was used, followed by Tukey’s multiple comparison test. The significance was considered when the *p*-value was less than 0.05 (*p* < 0.05).

## 3. Results

### 3.1. Methanol Extract Is Rich in Secondary Metabolites

**Figure 2:**
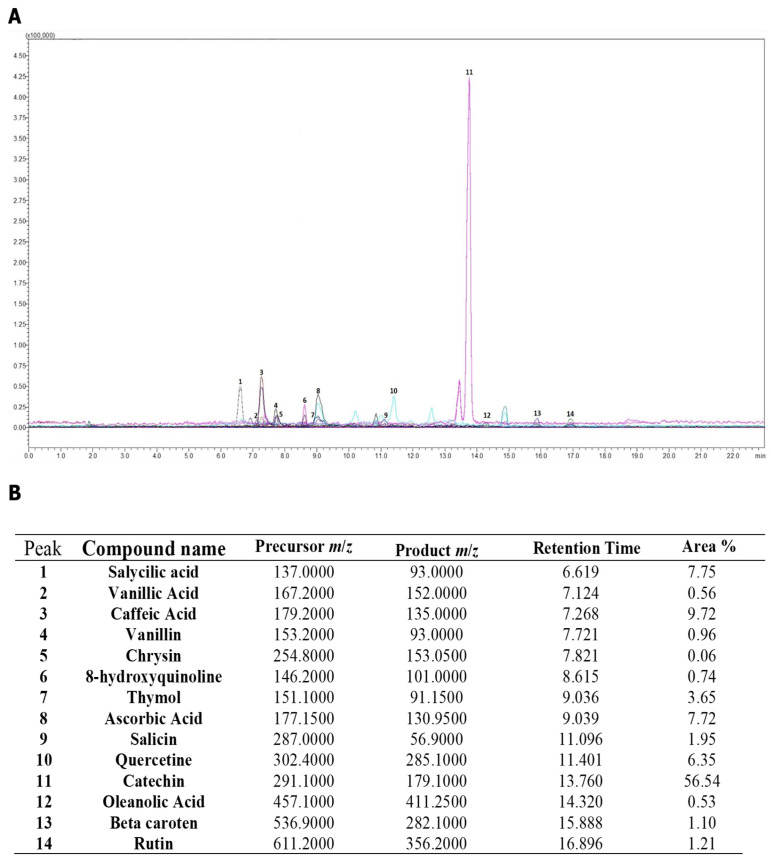
Ultra-performance liquid chromatography mass spectrometry (UPLC MS/MS) analysis of *Ranunculus repens* methanol extract (RRME). (**A**) UPLC MS/MS chromatogram of RRME; (**B**) summary of the compounds identified using UPLC-MS/MS. Each compound in the chromatogram is represented by a different color. The numbers indicated above each peak correspond to compound numbers listed in (**B**).

In order to identify the main components responsible for the various biological activities of RRME, the extract was analyzed using UPLC MS/MS to accurately determine its soluble compounds. The resulting chromatogram is shown in Figure 2A, and the corresponding compound data are detailed in Figure 2B. The analysis revealed that the majority of the detected compounds are phenolic compounds, mainly flavonoids. Among these, catechin was found to be the most abundant (56.54%), followed by caffeic acid (9.72%), salicylic acid (7.75%), ascorbic acid (7.72%), quercetin (6.35%), and thymol (3.65%).

### 3.2. RRME Reduced Pancreatic Acinar Damages in CP Model

**Figure 3:**
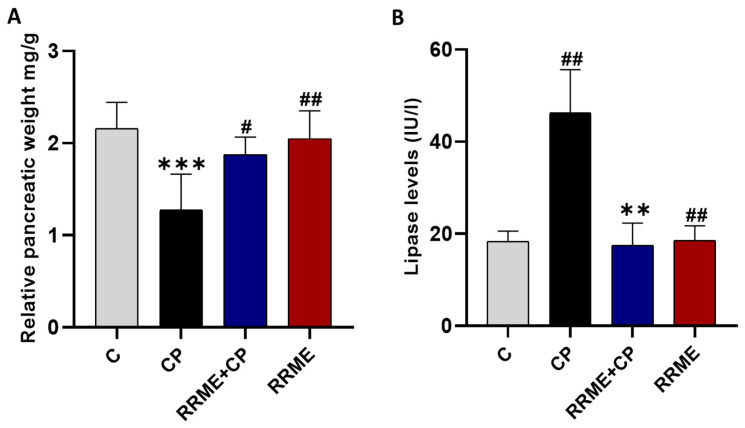
RRME mitigated pancreatic injury in the L-Arg-induced CP model. (**A**) Relative pancreatic weight (pancreas/body weight ratio); (**B**) Plasma lipase levels. RRME: *Ranunculus repens* methanol extract; CP: chronic pancreatitis; (C) Untreated group; (CP) Group treated with L-Arg only; (RRME + CP) Group treated with L-Arg and RRME; (RRME) Group treated with RRME only. Values are expressed as mean ± SD (*n* = 6). *** *p* < 0.001 and ** *p* < 0.01 significantly different from the control group; ## *p* < 0.01 and # *p* < 0.05 significantly different from CP group using one-way ANOVA followed by Tukey’s multiple comparison test.

Our results, presented in Figure 3A, showed that repeated administration of L-Arg led to severe pancreatic tissue damage. Furthermore, rats in the CP group exhibited a significant reduction in relative pancreatic weight due to progressive tissue atrophy compared with the control group (*p* < 0.001). However, treatment with RRME significantly improved the relative pancreatic weight in comparison to the CP group (*p* < 0.05; Figure 3A). Elevated levels of digestive enzymes in the serum are a hallmark of CP [4]. As shown in Figure 3B, the rats in the CP group exhibited a significant increase in lipase levels (*p* < 0.01) as compared to the control group. Furthermore, the treatment with RRME significantly decreased the levels of lipase compared to the CP group (*p* < 0.01).

### 3.3. RRME Attenuated Oxidative Stress in Pancreatic Tissues

**Figure 4:**
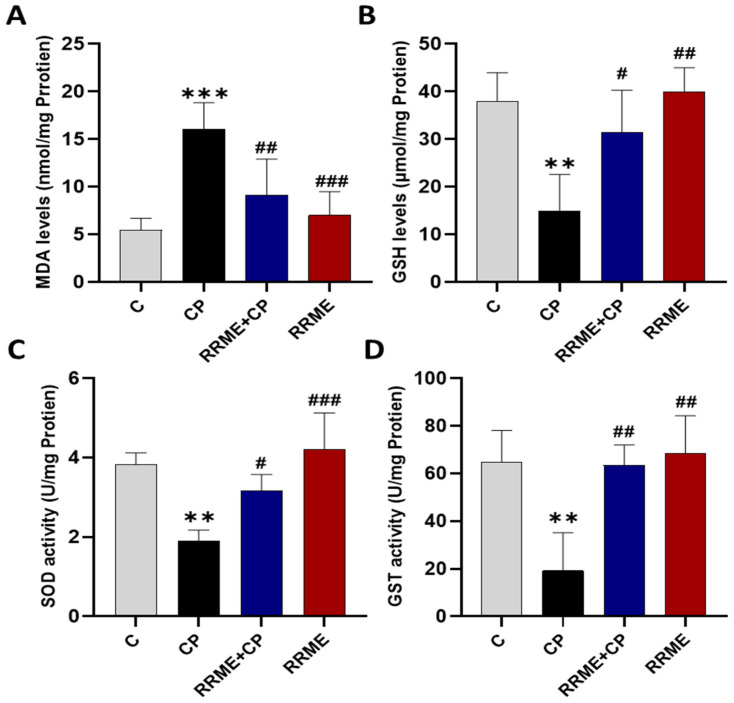
Effect of RRME on oxidative stress in pancreatic tissues. (**A**) Lipid peroxidation as represented by Malondialdehyde (MDA) levels; (**B**) Glutathione (GSH) levels; (**C**) Superoxide dismutase (SOD) activity; (**D**) Glutathione S-transferase (GST) activity. RRME: *Ranunculus repens* methanol extract; CP: chronic pancreatitis; (C) Untreated group; (CP) Group treated with L-Arg only; (RRME + CP) Group treated with L-Arg and RRME; (RRME) Group treated with RRME only. Values are expressed as mean ± SD (*n* = 5). *** *p* < 0.001, ** *p* < 0.01 and ### *p* < 0.001, ## *p* < 0.01 and # *p* < 0.05 significantly different from CP group using one-way ANOVA followed by Tukey’s multiple comparison test.

It is well established that OS plays a key role in the development and progression of CP. Therefore, the effect of RRME on OS markers in the pancreatic tissues of animal groups was examined. Lipid peroxidation is often used as an indicator of OS. As shown in Figure 4A, the CP group had significantly higher levels of lipid peroxidation in comparison to the control animals (*p* < 0.001). Furthermore, oral treatment with RRME significantly reduced the levels of lipid peroxidation (*p* < 0.01) compared to the CP group. OS is often associated with depletion of GSH content. Figure 4B shows a significant decrease in GSH content in the CP group (*p* < 0.01) in comparison to the control group. These low levels of GSH were significantly increased upon treatment with RRME (*p* < 0.05). Moreover, RRME treatment alone did not affect GSH content.

Our results also showed that treatment with RRME enhanced the enzymatic antioxidant system, as indicated by a significant increase in SOD activity compared to the CP group (*p* < 0.05), while the CP group exhibited a marked reduction in this activity compared to the control group (*p* < 0.01; Figure 4C). GST activity was also reduced in the CP group compared to the control group (*p* < 0.01). Treatment with RRME significantly elevated GST activity compared to the CP group (*p* < 0.05), as shown in Figure 4D. These results demonstrated the effectiveness of RRME in reducing OS in the CP experimental model.

### 3.4. RRME Reduced the Expression of Proinflammatory Cytokines TNF-α and IL-6

**Figure 5:**
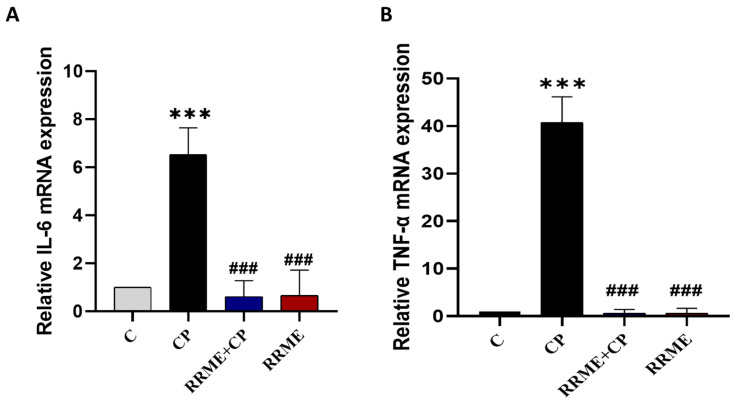
Effect RRME on the expression of proinflammatory cytokines. The mRNA expression of proinflammatory cytokines was analysed by RT-qPCR. (**A**) IL-6: Interleukin-6; (**B**) TNF-α: Tumour necrosis factor-alpha; RRME: *Ranunculus repens* methanol extract; CP: chronic pancreatitis (C) Untreated group; (CP) Group treated with L-Arg only; (RRME + CP) Group treated with L-Arg and RRME; (RRME) Group treated with RRME only. Values are expressed as mean ± SD (*n* = 3). *** *p* < 0.001 significantly different from control group; ### *p* < 0.001 significantly different from CP group using one-way ANOVA followed by Tukey’s multiple comparison test.

The critical role that proinflammatory cytokines TNF-α and IL-6 play in the progression of CP and the activation of PSCs is well established [53]. In the present study, RT-qPCR analysis showed that repeated administration of L-Arg caused a significant increase in the expression level of TNF-α by 40.71-fold compared to the control animals (*p* < 0.001), while treatment with RRME significantly suppressed TNF-α expression in pancreatic tissue reducing its expression by 66.73-fold (*p* < 0.001; Figure 5B). Furthermore, the animals in the CP group showed a significant increase in IL-6 expression by 6.54-fold relative to controls (*p* < 0.001). The elevated IL-6 expression was significantly reversed by RRME treatment by 10.72-fold (*p* < 0.001; Figure 5A). Additionally, no significant differences in TNF-α or IL-6 expression were observed between the animals that received RRME only and the control group.

### 3.5. RRME Down-Regulated mRNA Expression of Galectins 4 and 9 in CP

**Figure 6:**
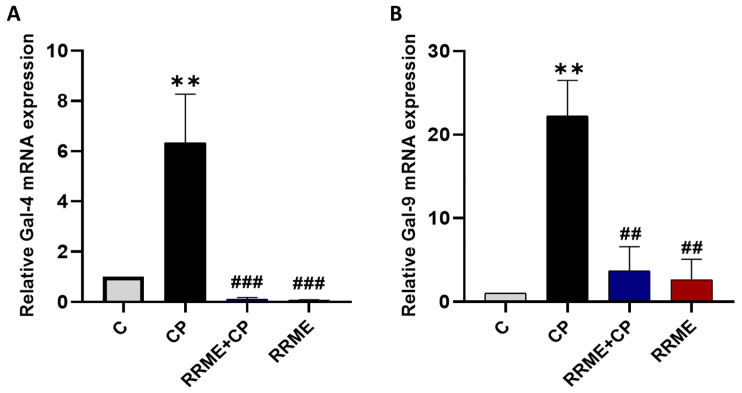
Effect of RRME on the expression of Gal-4 and Gal-9 mRNAs. The expression of Gal-4 and Gal-9 was analysed by RT-qPCR. (**A**) Gal-4: Galectin-4; (**B**) Gal-9: Galectin-9; RRME: *Ranunculus repens* methanol extract; CP: chronic pancreatitis (C) Untreated group; (CP) Group treated with L-Arg only; (RRME + CP) Group treated with L-Arg and RRME; (RRME) Group treated with RRME only. Values are expressed as mean ± SD (*n* = 3). ** *p* < 0.01 significantly different from the control group; ### *p* < 0.01 and ## *p* < 0.05 significantly different from CP group, using one-way ANOVA followed by Tukey’s multiple comparison test.

It is worth mentioning that in a previous work, we examined the relationship between Gal-9 and acute pancreatitis, as well as with RRME (work submitted for publication). Thus, we have already demonstrated that RRME significantly protected against L-Arg-induced AP, which appeared to be associated with the modulation of Gal-9 expression. The expression levels of Gal-4 and Gal-9 were evaluated by RT-qPCR. As shown in Figure 6, both Gal-4 and Gal-9 were significantly overexpressed in the CP group compared to the control group, with a fold increase of 6.33 for Gal-4 and 22.22 for Gal-9 (*p* < 0.01; Figure 6A and Figure 6B, respectively). Treatment with RRME significantly reduced the expression of Gal-4 by 63.3-fold (*p* < 0.001) and Gal-9 by 6-fold (*p* < 0.01) relative to the CP group. Notably, administration of RRME to normal animals had no effect on Gal-4 expression and caused a slight, non-significant increase in Gal-9 expression. The observed downregulation of Gal-4 and Gal-9 following RRME treatment correlated with a reduction in pancreatic injury and fibrosis, suggesting that these galectins play a role in the development or progression of CP.

### 3.6. RRME Reduced Collagen Deposition and Alleviated Histological Alterations

**Figure 7:**
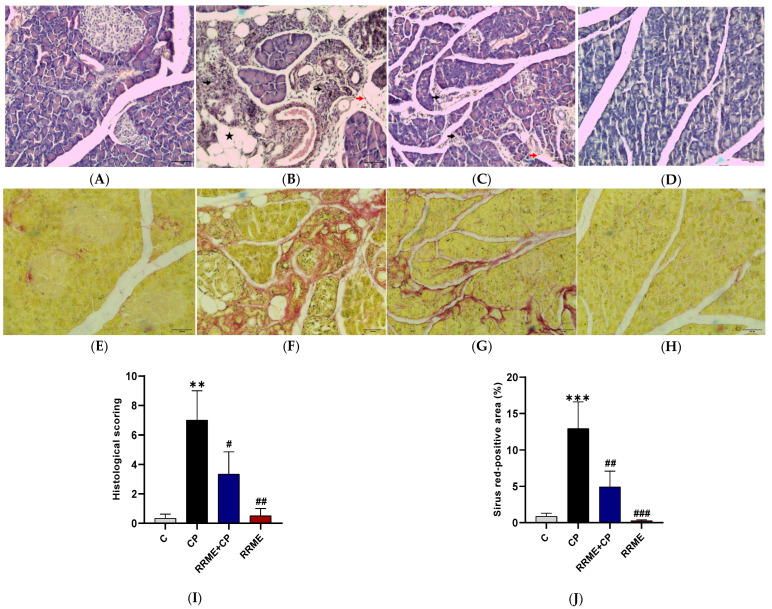
Effects RRME on pancreatic histology and collagen deposition. The pancreatic tissues from different groups of rats were stained with hematoxylin and eosin or picrosirius red staining. RRME: *Ranunculus repens* methanol extract; CP: chronic pancreatitis (**A**) Control group showed normal pancreatic architecture. (**B**) CP group showed significant glandular atrophy (black arrows), leukocyte infiltration (red arrows), and fat infiltration (black star). (**C**) RRME + CP group showed reduction in the severity of acinar atrophy. (**D**) RRME group showed typical pancreatic histology. (**E**) Control group showed minimal collagen deposition. (**F**) CP group showed high levels of collagen deposition around the duct system and within degenerated acini (red stained areas). (**G**) RRME + CP group showed an improvement in collagen deposition. (**H**) RRME group showed minimal collagen deposition. Magnification 10× Scale bars = 100 µM. (**I**) Histogram showing the effect of RRME treatment on histological scores of different experimental groups. (**J**) Histogram showing the quantitative analysis of the effect of RRME on collagen deposition across various experimental groups; (C) untreated group; (CP) group treated with L-Arg only; (RRME + CP) group treated with L-Arg and RRME; (RRME) group treated with RRME only. (Values are expressed as mean ± SD (*n* = 3). *** *p* < 0.001 and ** *p* < 0.01 significantly different from control group; ### *p* < 0.001, ## *p* < 0.01, and # *p* < 0.05 significantly different from CP group using one-way ANOVA followed by Tukey’s multiple comparison test.

Haematoxylin and eosin staining of pancreatic tissue sections showed that the animals in the CP group presented considerable histological alterations, including sublobular and lobular atrophy indicated by black arrows, fat infiltration indicated by a black star, and leukocyte infiltration indicated by red arrows (Figure 7B), which were reflected in a high histological score (Figure 7I). In contrast, tissue sections from the animal treated with the RRME displayed a histological architecture more similar to that of the control group (Figure 7C), with a significant reduction in glandular atrophy compared with the CP group (*p* < 0.05; Figure 7I). The animals treated with RRME alone and the control group exhibited normal histological features (Figure 7A,D).

The excessive deposition of ECM proteins, including collagens, contributes to the development of pancreatic fibrosis, which is one of the main pathological features of CP [54]. To investigate the effect of RRME treatment on the level of fibrosis in pancreatic tissues, picrosirius red staining was performed. Our results depict a significant increase in collagen deposition, represented by the red-stained area, especially in locations around degenerated pancreatic acini of the CP group (Figure 7F), in comparison to the control group (*p* < 0.001; Figure 7J). Importantly, treatment with RRME showed a substantial reduction in the levels of collagen depositions (*p* < 0.001; Figure 7G,J). Additionally, treatment of normal animals with RRME did not result in any rise in collagen deposition (Figure 7H).

### 3.7. RRME Reduced the Expression of Fibrosis-Related Genes in CP

**Figure 8:**
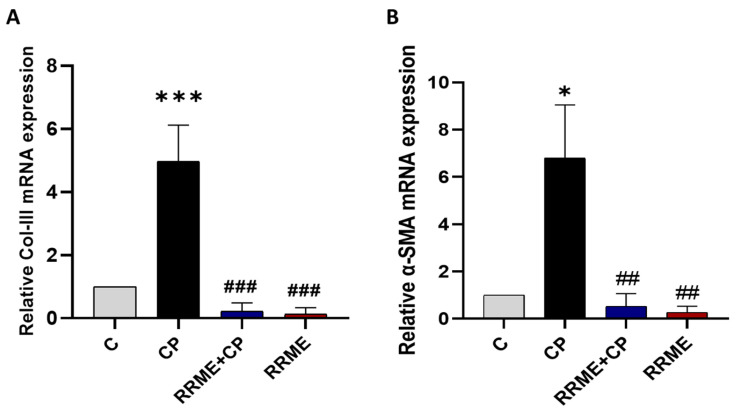
Effect of RRME on the mRNAs expression of fibrosis-related genes. The expression of fibrosis-related genes was analysed by RT-qPCR. (**A**) Col-III: collagen III; (**B**) α-SMA: alpha-smooth muscle actin; RRME: *Ranunculus repens* methanol extract; CP: chronic pancreatitis (C) Untreated group; (CP) Group treated with L-Arg only; (RRME + CP) Group treated with L-Arg and RRME; (RRME) Group treated with RRME only. Values are expressed as mean ± SD (*n* = 3). *** *p* < 0.001 and * *p* < 0.05 significantly different from the control group; ### *p* < 0.001 and ## *p* < 0.01 significantly different from CP group using one-way ANOVA followed by Tukey’s multiple comparison test.

We evaluated Col-III and α-SMA expression using RT-qPCR. Our results showed a clear increase in Col-III expression in the animals of the CP group by 4,7-fold as compared to the control counterpart (*p* < 0.001, Figure 8A). The increase in Col-III expression was significantly reversed by RRME treatment by 22.59-fold (*p* < 0.001). Moreover, α-SMA is a reliable marker for assessing PSCs activation in pancreatic tissues [55]. As presented in Figure 8B, the expression levels of α-SMA were noticeably increased by 6.8-fold in the CP group compared to the control animals (*p* < 0.05). Interestingly, α-SMA increased expression was decreased by 0.51-fold after RRME treatment as compared to the CP group (*p* < 0.01), suggesting that RRME treatment may have an impact on the activation of PSCs. Together, these results suggested that RRME may play a role in inhibiting the development of fibrosis during CP.

### 3.8. RRME Inhibited H_2_O_2_-Induced Oxidative Stress in Pancreatic Cell Lines

The MTT assay was performed to determine the non-toxic concentrations of RRME (Supplementary Figure S1) for subsequent use in the in vitro testing of its antioxidant ability. Thus, 10 and 25 µg/m RRME were selected, as both concentrations reduced cell viability by less than 25% after 72 h of treatment.

**Figure 9:**
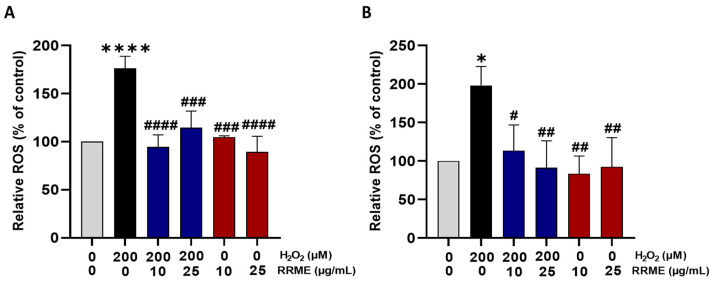
RRME suppressed OS in pancreatic cancer cells. (**A**) MiaPaCa-2 and (**B**) PANC-1 cells were evaluated by incubating them with DCF-DA. The cells were exposed to 200 µM of H_2_O_2_ and treated with either 10 µg/mL or 25 µg/mL of the extract for 24 h. RRME: *Ranunculus repens* methanol extract Values are expressed as mean ± SD (*n* = 3). **** *p* < 0.0001 and * *p* < 0.05 significantly different from the untreated cells; #### *p* < 0.0001, ### *p* < 0.001, ## *p* < 0.01 and # *p* < 0.05 significantly different from H_2_O_2_ treated cells using one-way ANOVA followed by Tukey’s multiple comparison test.

To evaluate the effect of the methanol extract on the levels of oxidative stress, we used DCFH-DA to label the cells. As shown in Figure 9, treatment with 200 µM H_2_O_2_ alone significantly increased OS in both pancreatic cell lines (*p* < 0.0001; Figure 9A) for MIA PaCa-2 and (*p* < 0.05; Figure 9B) for PANC-1. Treatment of the cells with the RRME at both concentrations (10 and 25 µg/mL) significantly inhibited the H_2_O_2_ induced OS in the MIA PaCa-2 cell line (*p* < 0.0001 for 10 µg/mL and *p* < 0.001 for 25 µg/mL). For the PANC-1 cell line, similar results were observed, with differences between the concentrations: *p* < 0.05 for 10 µg/mL and *p* < 0.01 for 25 µg/mL. However, treatment with the RRME at either 10 or 25 µg/mL did not significantly affect the level of OS in comparison to the control in either cell lines.

### 3.9. RRME Attenuated Paclitaxel-Induced Apoptosis in Mia PaCa-2 Cells

**Figure 10:**
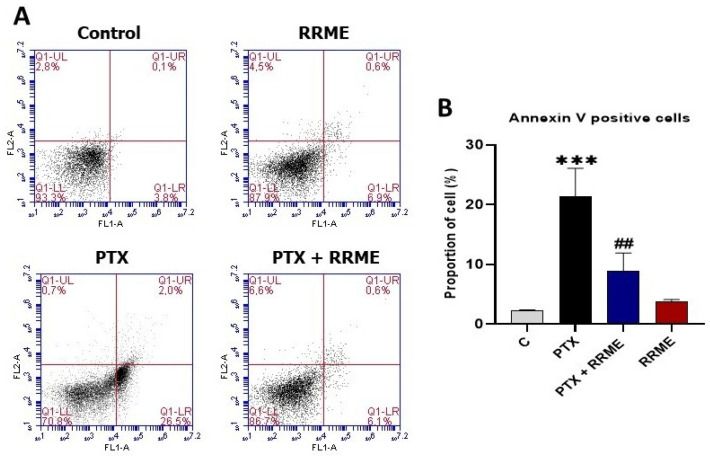
Effect of RRME on apoptosis in pancreatic cancer cells Mia PaCa-2 analysed using annexin V/PI staining. (RRME) Effect of *Ranunculus repens* methanol extract on apoptosis in Mia PaCa-2 cells; (PTX) Effect of paclitaxel alone on apoptosis in Mia PaCa-2 cells; (PTX + RRME) Effect of the combination of *R. repens* methanol extract and paclitaxel on apoptosis in Mia PaCa-2 cells; (Control) Untreated Mia PaCa-2 cells. (**A**) Flow cytometry dot plot showing cell distribution: (Q1) Upper left quadrant indicates necrotic cells, (Q2) Upper right quadrant indicates late apoptotic cells, (Q3) Lower left quadrant indicates viable cells, (Q4) Lower right quadrant indicates early apoptotic cells. (**B**) Histogram representing the percentage of cells positively stained for Annexin V. Values are expressed as mean ± SD (*n* = 3). *** *p* < 0.001 significantly different from the untreated cells; ## *p* < 0.01 significantly different from paclitaxel-treated cells using one-way ANOVA followed by Tukey’s multiple comparison test.

Paclitaxel is known to induce apoptosis in cancer cells [56]. Our flow cytometry analysis (Figure 10A) demonstrated that treatment of Mia PaCa-2 cells with paclitaxel significantly increased the proportion of Annexin V-positive apoptotic cells compared to the untreated control (*p* < 0.001; Figure 10B). Treatment with 100 µg/mL RRME (non-toxic for Mia Paca-2 cells) alone also led to a slight increase in apoptotic cells; however, this increase was not statistically significant. Interestingly, the combination of paclitaxel and RRME resulted in a significant reduction in apoptotic cell population compared to paclitaxel alone (*p* < 0.01), suggesting a potential protective effect of RRME against paclitaxel-induced apoptosis.

## 4. Discussion

CP is a progressive inflammatory disease that often develops following multiple episodes of pancreatic damage [57]. Each of these episodes is accompanied by the production of reactive oxygen species (ROS) [58], which leads to further acinar cell damage and the release of proinflammatory cytokines [59]. Sustained OS and inflammation eventually lead to the activation of PSCs and their differentiation into myofibroblasts, resulting in high production of ECM proteins and subsequent pancreatic fibrosis [10,54]. Given the burden that CP imposes on patients and the lack of suitable therapies, more effective alternative treatments must be explored. Natural compounds have significant therapeutic potential, including potent antioxidant activity. In recent years, plant-based bioactive compounds have attracted much attention as therapeutic agents to prevent or halt the progression of CP [60].

In the present study, we characterized the chemical composition of RRME using UPLC MS/MS and evaluated its antioxidant, anti-apoptotic, and anti-inflammatory effects, in both in vitro and in vivo models relevant to CP. Our findings demonstrate that RRME is rich in secondary bioactive compounds. Furthermore, it significantly reduced both OS and inflammation, two key drivers of pancreatic damage and fibrosis. In vitro RRME reduced intracellular ROS accumulation in response to H_2_O_2_ and decreased paclitaxel-induced apoptosis. Additionally, in the L-Arg-induced CP model, it attenuated the pancreatic damage, as evidenced by reduced histological alterations, pancreatic atrophy, and the progression of CP-associated fibrosis in pancreatic tissue. Moreover, it reduced OS and preserved the exocrine functions in the pancreas, as indicated by improved levels of the antioxidant markers and serum lipase. Additionally, RRME treatment lowered the expression of proinflammatory cytokines IL-6 and TNF-α. It may also affect the activation of PSCs, as suggested by decreased expression of α-SMA and Col-III. Notably, we reported for the first time that Gal-4 and Gal-9 mRNA levels were up-regulated in the CP L-Arg model compared to healthy controls, suggesting their potential as novel biomarkers for CP.

Our analysis of RRME using UPLC-MS/MS provided a deeper understanding of its chemical composition. Indeed, for the first time, several bioactive compounds were identified, including phenolic acids such as caffeic acid, vanillic acid, and salicylic acid, as well as various types of flavonoids such as quercetin (a flavonol), rutin (a flavonoid glycoside), catechin (a flavan-3-ol), and chrysin (a flavone). In addition, other phenolic compounds were detected, namely thymol (a terpenoid phenol), vanillin (a phenolic aldehyde), and oleanolic acid (a triterpenoid). Also, the extract contains β–carotene, a provitamin A, as well as ascorbic acid and salicin. Catechin is widely present in plants and has been shown to exhibit excellent antioxidant activity, comparable to that of ascorbic acid and glutathione [61], as well as anti-inflammatory properties [62,63]. Caffeic acid is a naturally occurring polyphenolic compound present in different species of the Ranunculaceae family, including *R. arvensis* [34], *R. macrophyllus* [35], and *R. japonicus* [64]. It exhibits several biological activities, such as reducing inflammation and oxidative stress [65]. Quercetin is a flavonol present in *R. arvensis* [34], *R. macrophyllus* [35] and *R. bulumei* [36]. However, the number of identified compounds was limited by the availability of standards, and additional bioactive constituents may be detected through more comprehensive or advanced analyses in future studies. Furthermore, the data presented in this study are qualitative rather than quantitative; therefore, more advanced analyses using calibration curves should be conducted to quantify the detected bioactive compounds.

One of the main clinical observations in patients with CP is the reduction in pancreatic size [66]. Our findings showed that multiple administrations of L-Arg caused significant pancreatic damage, clearly manifested by the reduction in pancreatic relative weight in the CP group, alongside histopathological features that resemble CP, like inflammatory cell infiltration, glandular atrophy, fatty infiltration, and fibrosis. However, we acknowledge that this model does not reproduce all aspects of human chronic pancreatitis, and features such as fatty infiltration occur only in rare cases in patients.

Furthermore, the increase in lipase activity is considered one of CP markers [67], this remark supports our results that reported elevated activities of serum lipase in the CP group, which can be attributed to acinar cell death and damage, leading to elevated levels of digestive enzymes. These features have also been observed in animal models of CP by Balaha et al. [68]. Our results showed that RRME treatment significantly improved relative pancreatic weight and reduced. A Comparable effect on the relative pancreatic weight was also documented using natural bioactive compounds such as Berberine in the context of cerulein-induced CP [69]. Moreover, the mitigation of pancreatic injury was also demonstrated by various histopathological changes, including decreased inflammatory cell infiltration, reduced glandular atrophy, and less fibrosis in pancreatic tissues. These observations are consistent with reports on herbal decoctions made using traditional herbs such as the Dachaihu decoction, which was shown to reduce leukocyte infiltrations, acinar necrosis, as well as pancreatic fibrosis [70]. Parallel to this, it improved pancreatic exocrine function, as evidenced by the significant reduction in serum lipase activity. A similar effect on serum lipase was observed using olive leaf extract [71]. These effects of RRME are consistent with those produced by natural antioxidants and plant extracts in CP experimental models [68,72,73].

The pancreas is particularly susceptible to prolonged OS in comparison to other organs like the kidney or the liver [74], which is considered one of the main drivers of CP progression [3], promoting pancreatic injury and creating a vicious cycle that amplifies the inflammatory response [59]. we considered it relevant to use PANC-1 and Mia PaCa-2 cell lines to study the effects of RRME extract in vitro. Although these two cell lines are derived from the exocrine compartment of the pancreas, they are cancer-derived and therefore do not fully recapitulate the biology of normal acinar or pancreatic stellate cells involved in CP. Nonetheless, they are widely used to study OS and evaluate the antioxidant effects of natural compounds [75]. Interestingly, our study demonstrated that treatment with RRME suppressed H_2_O_2_-induced OS in both MIA PaCa-2 and PANC-1 cell lines.

These findings are consistent with those of previous studies, which reported antioxidant properties of *R. repens* leaves [42]. These last ones were demonstrated both through the scavenging of DPPH free radicals in vitro and the alloxan-induced animal model. We also investigated the effect of RRME on OS in pancreatic tissues. Notably, we found that RRME reduced OS, as evidenced by the preservation of GSH content and the mitigation of lipid peroxidation, as indicated by lower MDA levels. Furthermore, it enhanced the activities of SOD and GST. Similar antioxidant effects have been reported using natural extracts and plant-based bioactive compounds [44,68,71]. This antioxidant effect of RRME may be attributed to the presence of high levels of phenolic compounds and flavonoids in leaves of *R. repens* which include several powerful anti-oxidants such as catechin, caffeic acid, rutin, and chrysin [61,76,77,78]. Furthermore, the plants of *Ranunculus* species are well known for their high phenolic content, Deghima et al. [35] detected high phenolic content in *Ranunculus macrophyllus*, as these bioactive compounds are known to possess potent antioxidant activities [79]. However, to definitively establish the contribution of these specific compounds to the antioxidant activity of RRME, further isolation, characterization, and targeted bioactivity testing are required. In contrast, the CP group exhibited high levels of OS, manifested by GSH depletion, elevated lipid peroxidation and significantly lower SOD and GST activities. These results are similar to those of several previous works [51,80,81]. In addition, OS triggers the activation of PSCs [82], and the inhibition of SOD by diethyldithiocarbamate, eventually leads to the development of pancreatic fibrosis [74].

OS not only exacerbates pancreatic inflammation, but also induces apoptosis in pancreatic cells [68]. The number of apoptotic cells significantly increases during chronic pancreatitis, contributing to the destruction and loss of acinar cells [83]. Our results demonstrated that treatment of Mia PaCa-2 cells with the RRME reduced paclitaxel-induced apoptosis. Zhang et al. [84] reported that paclitaxel-induced apoptosis in cancer cells is mediated by an increase in intracellular ROS accumulation. Interestingly, our findings revealed that treatment with the RRME significantly reduced intracellular ROS levels, suggesting that the extract may mitigate paclitaxel-induced apoptosis by alleviating OS. This antioxidant property of the RRME suggested a broader protective effect. Therefore, the ability of RRME to mitigate OS may underlie its protective effect against the progression of CP.

The prolonged OS and the production of high levels of proinflammatory cytokines such as TNF-α and IL-6 lead to the activation of PSCs. When activated, PSCs are characterized by the expression of α-SMA [55]. In an inflammatory environment, PSCs remain activated by various exogenous and endogenous signals and contribute to the progression of CP by excessively producing collagen and other ECM proteins, ultimately leading to pancreatic fibrosis [72].

In our study, multiple administrations of L-Arg resulted in a remarkable up-expression of IL-6, TNF-α and α-SMA, this is supported by data from relatively recent works [68,80]. Indeed, the overexpression of α-SMA in CP animal models and in human CP has been documented in several studies [11,44,85]. Our results also showed that RRME treatment significantly reduced the expression of IL-6, TNF-α and α-SMA, indicating its strong anti-inflammatory potential. Similar results were reported using plant-based bioactive compounds. For instance, fraxetin significantly reduced the levels of both IL-6 and TNF-α in a CP animal model, while tocotrienols attenuated α-SMA expression in an L-Arg–induced CP model, demonstrating an anti-fibrotic effect. Likewise, the Dachaihu decoction was shown to decrease IL-6 expression and mitigate pancreatic inflammation [44,68,70]. Pancreatic fibrosis is a major hallmark of CP [4]. Furthermore, plant-based bioactive compounds have been shown to exert potent antifibrotic effects by mitigating the role of PSCs in CP through several mechanisms [31]. Our findings indicated a clear reduction in pancreatic fibrosis after treatment with RRME, as reflected by the decrease in both deposition (picrosirius red staining) and expression (RT-qPCR) of Col-III. These results are consistent with previous studies showing that mitigating inflammation, ECM proteins production, and PSCs activation reduces pancreatic fibrosis and disease progression [11,81].

In contrast, animals in the CP group had substantially higher levels of fibrosis in their pancreatic tissue, which was also reported by Bava et al. [86]. The CP group also exhibited high levels of Col-III expression, which has been reported as well by Li et al. [81]. Furthermore, relatively recent studies have reported the anti-inflammatory effects of several bioactive compounds present in RRME, such as quercetin, caffeic acid, and catechin. These compounds have been shown to exert beneficial effects in both acute and chronic pancreatitis [87,88,89]. Thus, we speculate that these compounds contribute significantly to the protective effects of RRME against L-Arg induced CP.

It is worth remembering that Gal-4 and Gal-9 both belong to the subgroup of galectins possessing two CRDs and have been reported to play different roles in various physiological and pathological processes [90,91]. Regarding Gal-4, it was shown to exacerbate intestinal inflammation, leading to the production of the inflammatory cytokine IL-6 [24]. On the other hand, Paclik et al. [25] reported that Gal-4 played a regulatory role in intestinal inflammation, reducing the secretion of TNF-α, IL-6, and IL-8. Moreover, the mRNA of this protein was reported to be down-regulated in colorectal cancer [92] and its abrogation led to the promotion of tumorigenesis as well as the up-regulation of IL-6 [93]. Additionally, it is over-expressed in pancreatic cancer [94] and this over-expression has been associated with reduced metastatic potential in pancreatic ductal adenocarcinoma [22,95].

In parallel, Gal-9 is involved in inflammatory activity; indeed, its level was increased in the plasma of patients with rheumatoid arthritis, and its neutralization in fibroblast-like synoviocytic cells of these patients led to a decrease in the production of IL-6 [96]. Moreover, it is detected in human pancreatic ductal adenocarcinoma [23] and its neutralization by monoclonal antibody protected against tumour progression and prolonged survival rates in an animal model of pancreatic ductal adenocarcinoma [97]. In our study, we found that the expression of both Gal-4 and Gal-9 was very high in animals with CP, while RRME treatment significantly reduced their expression, and this was correlated with a marked improvement in pancreatic injury and inflammation. However, as our analysis was limited to the transcriptional level, further validation at the protein level using analyses, such as Western blotting or immunohistochemistry, would strengthen the evidence that Gal-4 and Gal-9 are overexpressed in CP and that RRME directly modulates their expression. Moreover, investigating the molecular pathways linking RRME to galectin regulation could clarify its mechanistic role in inflammation and fibrosis.

## 5. Conclusions

In total, all the results obtained in the present study demonstrated that RRME is rich in secondary metabolites. Additionally, it effectively suppressed H_2_O_2_-induced intracellular ROS production and significantly reduced paclitaxel-induced apoptosis in vitro. Furthermore, RRME inhibited the progression of experimental CP and pancreatic fibrosis through attenuation of OS and protection against acinar cell death and glandular atrophy, as well as reduction in PSC activation. Moreover, this study is the first to show that Gal-4 and Gal-9 were overexpressed in L-Arg-induced CP and that RRME affected their expression levels. However, further studies are required to deepen these findings and elucidate the molecular mechanisms through which RRME inhibits PSC activation. Future research should focus on isolating and characterizing the individual bioactive compounds responsible for the observed effects, as well as evaluating their possible synergistic interactions. Validation of galectin-4 and galectin-9 modulation at the protein level is also essential to confirm their potential as biomarkers or therapeutic targets in chronic pancreatitis. Moreover, their precise roles in the pathogenesis of CP should be clarified through additional mechanistic studies. Considering the antifibrotic effects of RRME in chronic pancreatitis, it would also be valuable to explore its modulatory influence within the pancreatic cancer microenvironment using both cellular and animal models. Such an approach is likely to open relevant perspectives for the diagnosis and/or treatment of this serious disease.

## Figures and Tables

**Table 1:** Primers sequences used for RT-qPCR.

Gene	Accession Number	Forward Primer (5′-3′)	Reverse Primer (5′-3′)
*Lgals4*	NM_012975.2	ATCCCAGTATCCGGGAACCA	ACAGGCGGATTGAAGATCGG
*Lgals9*	NM_001388499.1	TGGACACCATTTCGGTCTCG	AAAGCCCTGAGTTCGCTGTT
*Tnf*	NM_012675.3	GATCGGTCCCAACAAGGAGG	TCCGCTTGGTGGTTTGCTAC
*IL-6*	NM_012589.2	CCCACCAGGAACGAAAGTCAA	TGGCTGGAAGTCTCTTGCGG
*Acta2*	NM_031004.2	CTATGCTCTGCCTCATGCCA	CTCACGCTCAGCAGTAGTCA
*Col3a1*	NM_032085.1	GGCCTCAAGGTGTAAAGGG	GGGCCCTGGATTACCATTGTT
*Gapdh*	NM_017008.4	GCCGCATCTTCTTGTGCAGT	CGATACGGCCAAATCCGTTCA

## Data Availability

The original contributions presented in this study are included in the article/Supplementary Materials. Further inquiries can be directed to the corresponding authors.

## Supplementary Materials

The following supporting information can be downloaded at: https://www.mdpi.com/article/10.3390/antiox14121436/s1, Figure S1: Effect of *R. repens* methanol extract on cells viability in pancreatic cancer cell lines PANC-1 and MiaPaCa-2, assessed using the MTT assay; Figure S2: Effect of *R. repens* methanol extract on cell viability in mouse pancreatic beta cells NIT1, assessed using the XTT assay.
