# Supplementary material for: Methanol Extract from Ranunculus repens L. Down-Regulated Galectins 4 and 9, and Mitigated Chronic Pancreatitis in an Experimental Rat Model
Source: Antioxidants (Basel). 2025 Nov 28;14(12):1436. doi: 10.3390/antiox14121436 (PMC12729588; doi:10.3390/antiox14121436)
Supplement: Supplementary file 1 [file antioxidants-14-01436-s001.zip › antioxidants-3923315-Figures S1 and S2.pdf]

S1. Supplementary Materials and Methods

S1.1 MTT Assay

MTT assay was performed as previously reported [1]. Briefly, cells (2000 cells/well) were seeded in 100 µl of serum-supplemented medium and treated with different concentrations of *RRME*. Untreated cells were used as control. After 72 h, the viability was assessed by adding MTT to control and treated cells to a final concentration of 0.5 mg/ml for 2 h. At the end of this period, the medium was removed, 100 µl of DMSO were added and the absorbance was recorded at 530 nm by a 96-well-plate ELISA reader.

S1.2 XTT Assay

To evaluate the cytotoxic effect of RRME on NIT cells, a total of 1,000 cells per well were seeded in 190 µl of serum-supplemented medium. After 24 hours, the cells were treated with different concentrations of RRME, while untreated cells were used as controls. The plate was then incubated for 72 hours. Afterwards, the culture medium was removed, the wells were washed with PBS, and 200 µl of fresh medium was added. The XTT1 and XTT2 reagents from the XTT kit were mixed, and 50 µl of this mixture was added to each well. After 4 hours of incubation, absorbance was measured at 450–500 nm using an automatic plate reader.

S2. Supplementary Results

S2.1 Effect of RRME on the Viability of PANC-1 and MiaPaCa-2 Cells

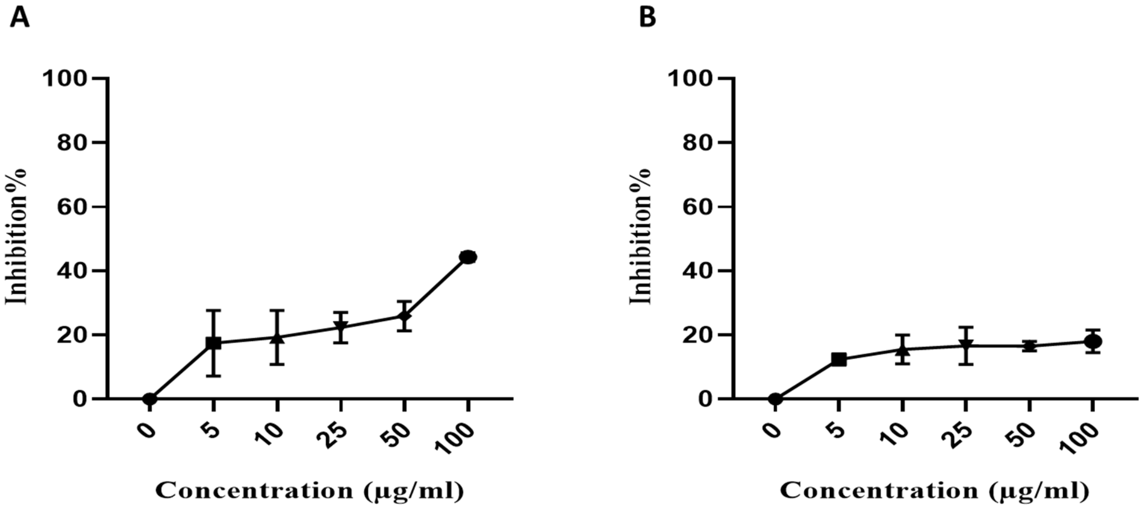

Figure S1. Effect of *R. repens* methanol extract on cells viability in pancreatic cancer cell lines PANC-1 and MiaPaCa-2, assessed using the MTT assay. (**A**) PANC-1 cells and (**B**) MiaPaCa-2 cells were treated with increasing concentrations (5–100 µg/mL) of the *R. repens* methanol extract for 72 h. Values are expressed as mean ± S.D (n = 3).

S2.2 Effect of RRME on the Viability of NIT1 Cells

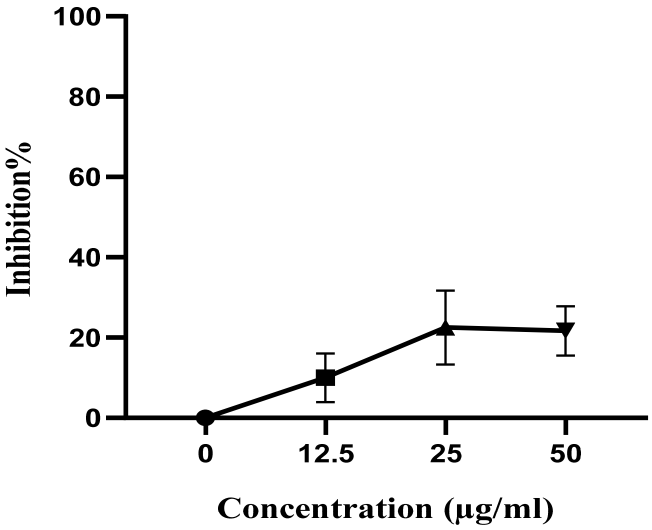

Figure S2. Effect of *R. repens* methanol extract on cell viability in **mouse pancreatic beta cells NIT1**, assessed using the XTT assay. NIT cells were treated with increasing concentrations (12.5–50 µg/mL) of the *R. repens* methanol extract for 72 h. Values are expressed as mean ± S.D (n = 3).

Reference

1. Ellman, G.L. Tissue sulfhydryl groups. *Arch. Biochem. Biophys.* **1959**, *82*, 70–77.
